# Neural Plastic Effects of Cognitive Training on Aging Brain

**DOI:** 10.1155/2015/535618

**Published:** 2015-08-31

**Authors:** Natalie T. Y. Leung, Helena M. K. Tam, Leung W. Chu, Timothy C. Y. Kwok, Felix Chan, Linda C. W. Lam, Jean Woo, Tatia M. C. Lee

**Affiliations:** ^1^Laboratory of Neuropsychology, The University of Hong Kong, Hong Kong; ^2^Laboratory of Social Cognitive Affective Neuroscience, The University of Hong Kong, Hong Kong; ^3^Institute of Clinical Neuropsychology, The University of Hong Kong, Hong Kong; ^4^Department of Medicine, The University of Hong Kong, Hong Kong; ^5^Alzheimer's Disease Research Network, The University of Hong Kong, Hong Kong; ^6^Department of Medicine and Therapeutics, The Chinese University of Hong Kong, Hong Kong; ^7^Fung Yiu King Hospital, Hong Kong; ^8^Department of Psychiatry, The Chinese University of Hong Kong, Hong Kong; ^9^State Key Laboratory of Brain and Cognitive Science, Li Ka Shing Faculty of Medicine, The University of Hong Kong, Hong Kong

## Abstract

Increasing research has evidenced that our brain retains a capacity to change in response to experience until late adulthood. This implies that cognitive training can possibly ameliorate age-associated cognitive decline by inducing training-specific neural plastic changes at both neural and behavioral levels. This longitudinal study examined the behavioral effects of a systematic thirteen-week cognitive training program on attention and working memory of older adults who were at risk of cognitive decline. These older adults were randomly assigned to the Cognitive Training Group (*n* = 109) and the Active Control Group (*n* = 100). Findings clearly indicated that training induced improvement in auditory and visual-spatial attention and working memory. The training effect was specific to the experience provided because no significant difference in verbal and visual-spatial memory between the two groups was observed. This pattern of findings is consistent with the prediction and the principle of experience-dependent neuroplasticity. Findings of our study provided further support to the notion that the neural plastic potential continues until older age. The baseline cognitive status did not correlate with pre- versus posttraining changes to any cognitive variables studied, suggesting that the initial cognitive status may not limit the neuroplastic potential of the brain at an old age.

## 1. Introduction

Neural plasticity refers to the capacity of our brain to change in response to internal demand and/or external experience [1]. Burgeoning research has corroborated that the neural plastic changes induced in our brains and behaviors are specific to the experiences (e.g., [2–7]). For instance, the London taxi drivers who have protracted experience in driving around the city with complex road infrastructure demonstrated significant increases in brain structural volume in the posterior hippocampus, which is implicated in storing spatial representation of the environment, suggesting that intensive experiences with spatial navigation can induce specific neural plastic changes in the corresponding brain region [3]. Similarly, Lee et al. [6] have identified distinct patterns of neural activation associated with different forms of meditation practice, namely, focused-attention meditation (FAM) and loving-kindness meditation (LKM), during the performance of the sustained attention task. While FAM is a form of meditation practice with a heavy emphasis on focusing attention on a particular object [8], LKM stresses on the cultivation of a state of universal love and compassion as to relieve pain and suffering for the self and others [9]. Their findings revealed that FAM practitioners (both experts and novices) showed significantly greater neural activation in the attention-related network when engaging in the sustained attention task, whereas similar neural activation was not observed in the LKM practitioners. The dissociable neural pattern associated with FAM and LKM indicated that meditation, as a form of mental exercise, could induce domain-specific neural plastic changes that accord with the form of meditation practice [6]. These findings offer an insight that perhaps a cognitive experience can be designed to specifically target triggering experience-dependent neural plastic changes in the cognitive domain of interest.

Yet, our brain, albeit malleable, inevitably undergoes certain degrees of age-associated cortical degeneration at the molecular level as age advances. For instance, significant reduction in global and regional gray matter volume, particularly in the prefrontal and medial temporal regions (e.g., [10–13]), as well as age-related alterations in the functional connectivity of the default mode network (e.g., [14, 15]) have been consistently reported in older adults. These age-related neural changes may cause cognitive deterioration in the aging brain.

Substantial research has yielded evidence of age-related decrements in attention and working memory performance (e.g., [16–20]). Mani et al. [21] observed that older adults committed significantly more errors than younger adults when performing a task on sustained attention, suggesting that the ability to sustain attention (in terms of response accuracy) was compromised during the natural aging process. It has been speculated that older adults, in order to compensate for their deficits in attentional performance, are required to recruit additional cognitive resources [20, 22]. Working memory also declines with aging. Mattay et al. [23] observed that the performance of older relative to younger adults declined with increased working memory load. In terms of long-term memory, profound changes in episodic memory have also been evidenced [24, 25], which can be attributed to the age-associated degeneration in the medial temporal regions, especially the hippocampus. Findings from a six-year longitudinal study revealed that hippocampal atrophy and a reduction of neural activation in the hippocampus were only identified in older adults demonstrating declines in memory performance but not in those with intact memory function [26]. Findings of all these studies highlight the natural course of cognitive degeneration with aging. Research on neuroplasticity-based intervention for age-associated cognitive changes is timely.

Despite the deleterious effects of age on the brain and behavior, increasing research has shown that the neural plastic potential is preserved until late adulthood (e.g., [27]). Cabeza [28] found that high-performing older adults could counteract age-associated neural decline by recruiting bilateral prefrontal regions when performing a cognitively demanding source memory task, as opposed to the right lateralization observed in both younger adults and low-performing older adults. The reduction in hemispheric asymmetry appears to compensate for the age-associated decline in cognitive performance by reorganizing the neural recruitment [28, 29]. In another study, Boyke et al. [30] found that older adults demonstrated a similar extent of gray matter changes in the middle temporal area of the visual cortex (hMT/V5) as did their younger counterparts, after acquiring the skill of juggling a three-ball cascade. Although the juggling performance of older adults was less proficient than that of their younger counterparts, the observed brain structural changes in the visuomotor region bolstered up the claim that an aging brain is still capable of change in response to experience. Further support was substantiated by Colcombe et al. [31], which showed that older adults demonstrated significant increases in brain structural volume after completing aerobic exercise training, in comparison to that of the older adults in the stretching control group.

Considering that our brain retains its neural plastic potential until late adulthood, lately there has been a growing trend toward exploring any cost-effective intervention that can mitigate or even slow down the age-related declines in cognitive functions. Such cognitive training or interventions are primarily targeted at training either general cognitive function or specific cognitive domains, such as attention and working memory, and promising findings have been received thus far (e.g., [32–35]). Building on the principle of neuroplasticity and the concept of the sensory deprivation model, Smith et al. [36] developed a self-administered cognitive training program that aimed at improving the auditory processing speed as well as its accuracy. Their program consists of six computerized exercises that stress sharpening one's ability to discriminate, judge, recognize, and match the sequences or pairs of confusable syllables; to reconstruct the sequences of verbal instruction; and to discern the details after listening to a story presented verbally. Individuals are encouraged to perform cognitive exercises for one hour every day, five days per week, for a total of eight weeks (equivalent to 40 sessions). It was found that participants demonstrated significantly greater improvement in auditory attention and memory after completing the Brain Fitness Program, when compared with their peers in the Active Control Group [36].

Recently, the feasibility and potential of implementing the cognitive training program to older adults at risk of cognitive decline have garnered increasing empirical attention and equivocal evidence has been obtained (e.g., [37, 38]). It is important to gather evidence on whether the cognitive training can induce general or specific neural plastic changes in cognitive function among the older adults who experience greater than normal rate of age-related cognitive decline and predispose to greater risk of developing dementia or specifically AD (e.g., [39, 40]). To bridge the aforementioned research gap, the current study was a longitudinal study that examined the specific cognitive effects of planned experience delivered in a cognitive training program. The program, modeled after the Brain Fitness Program [36], aimed at providing the training for attention and working memory but not verbal or visual-spatial memory of older adults at risk of cognitive decline. Based on the principle of experience-induced neural plasticity, it is hypothesized that participants from the Cognitive Training (CT) Group, relative to their peers in the Active Control (AC) Group, would demonstrate training-specific improvement in attention and working memory but not verbal and visual-spatial memory following 13 weeks of cognitive training.

## 2. Method

### 2.1. Participants

Ethics approval of this study was granted by the Institutional Review Board of the University of Hong Kong/Hospital Authority Hong Kong West Cluster (HKU/HA HKW IRB). Informed consents for participation in this study were obtained from the 209 right-handed community-dwelling Chinese older adults at risk of cognitive decline recruited via a local monthly newsletter, local elderly centers, and word of mouth. These older adults were considered at risk of cognitive decline because their Montreal Cognitive Assessment (MoCA) scores fell into the range of 19 to 26 [41]. Other than meeting the MoCA's cutoff score, the participants also fulfilled the following inclusion criteria: they (1) were 60 years old or above, (2) were literate, (3) had normal or corrected-to-normal vision and/or hearing, (4) were right-hand dominant as assessed by the Lateral Dominance Test [42], and (5) had normal intelligence as measured by the Test of Nonverbal Intelligence (TONI-III) [43]. Participants were excluded from this study if they met one of the criteria below: they (1) had current or a history of neurological or psychological disorders (e.g., head injuries, stroke, major depression, or generalized anxiety disorder), (2) had current or a history of substance abuse and/or alcoholism, (3) were on antidementia medication and/or (4) were diagnosed with thyroid dysfunction or vitamin B12 deficiency, and (5) scored in the moderate or severe range (≥11) in either subscales (depression or anxiety) of Hospital Anxiety and Depression Scale (HADS) [44].

The 209 participants were randomly assigned to the CT and AC groups by an experimenter blind to the cognitive status of the participants using computer-generated random sequences of numbers. Specifically, each participant ID was paired with a random number and the order of the participants was rearranged based on the value of the assigned number (from smallest to largest). Results of Significance Test confirmed that the CT and AC groups did not differ in demographic characteristics, cognitive processing speed, and depression scores, variables that have been consistently reported to play a significant role in modulating the rate of cognitive decline (e.g., [45–48]).

### 2.2. Cognitive Training: Training Protocol

The training protocol used in this study was modelled after the Brain Fitness Program (Posit Science, San Francisco, California, Glenn Smith). It is a self-administered training program of three one-hour sessions per week for a total of thirteen weeks. Older participants in the CT group were encouraged to practice four out of six cognitive exercises (approximately 15 minutes each) in each training session. In the following, a brief description of each of the six cognitive exercises was provided.

#### 2.2.1. Sound Sweeps

In each trial, participants are presented with two sound sweeps. Each sound sweep either begins low and rises upward or begins high and goes downward. Participants are instructed to indicate the frequency of the two sound sweeps by clicking the corresponding arrows using the mouse. For example, up-arrow “↑↑” represents that the frequency of the sound sweep shifts from low to high, whereas the down-arrow “↓↓” means that the frequency of the sound sweep shifts from high to low. Participants have to respond as quickly and accurately as they can. Their reaction times and response accuracy for each trial are recorded.

#### 2.2.2. Size Discrimination

In this exercise, participants will be presented with two orange heptagons in each trial. Their task is to discriminate between the different sizes of the heptagons and to identify which heptagon (left or right) has a relatively larger size. To increase the level of difficulty, the size contrast decreases (i.e., the size difference is not that apparent) as the number of trials advances. Their reaction time and response accuracy for each trial are recorded.

#### 2.2.3. Matching Pairs of Syllables

In each trial, eight cards are shown on a screen. Each card is associated with a syllable. Whenever the participants click on each card, they hear a specific sound (syllable). Their task is to pair up the same syllables that are randomly dispersed among the eight cards. Participants can hear the sound as many times as they can but they are encouraged to complete the matching task using the minimal number of steps. Their reaction times and response accuracy for each trial are recorded.

#### 2.2.4. Matching Pairs of Rhythm

In each trial, participants hear two pairs of rhythm and are asked to recognize whether the two pairs of rhythm are the “same” or “different.” The level of difficulty increases with increased length of the rhythm. The participants' reaction times and response accuracy for each trial are recorded.

#### 2.2.5. Chasing the Stars

In this exercise, participants are first shown three stars appearing in random locations on the screen in quick succession. Their task is to repeat the sequence and location where the three stars were shown on the screen in the same order. If the participants' performance reaches a certain level of accuracy, they would proceed with “four-star” and “five-star” conditions. Their reaction times and response accuracy for each trial are recorded.

#### 2.2.6. Narrative Stories

Participants are instructed to listen to a number of short narrative stories that comprise short conversations and answer the questions pertaining to the details of the conversation afterward.

### 2.3. Active Control

The purpose of the AC group was to ascertain whether the observed improvement in cognitive functioning can be attributed to factors (e.g., the training format) other than the training content. To account for the confounding factors, the training duration, frequency, and format of the AC group were comparable to those of the CT group, except the content of the training. Participants in the AC group did not work on the same kinds of cognitive exercises as did their peers in the CT group. Instead, they were shown educational programs covering diverse topics (e.g., history, science, health information, and local social issues) on a group basis. Immediately after watching the video, they were instructed to answer several questions that were related to the video content.

### 2.4. Study Design and Procedure

**Figure 1:**
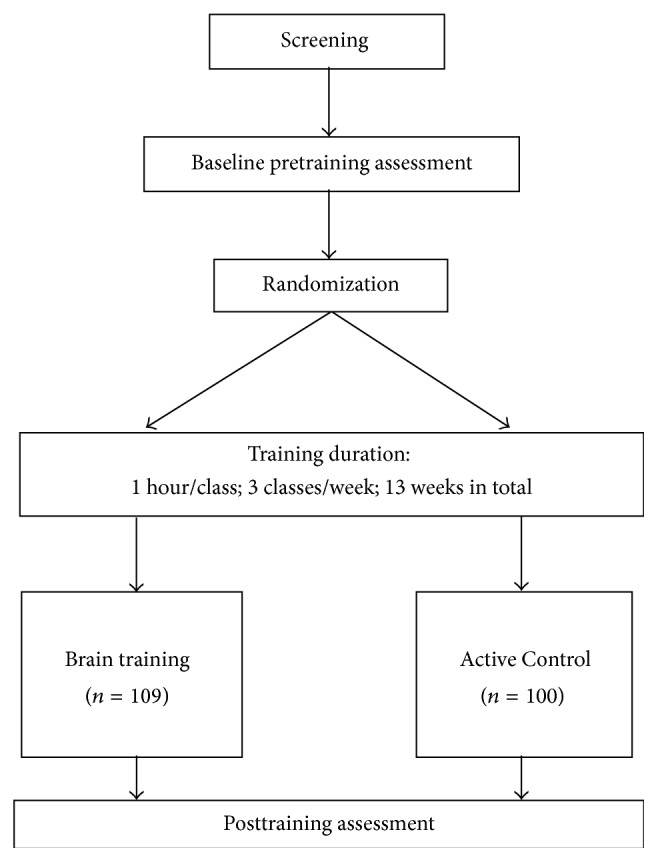
Flowchart of the longitudinal study of the thirteen-week cognitive training program.

The current study adopted a longitudinal study design to evaluate the effects of a brain plasticity-based training program on enhancing attention and working memory in a group of Chinese older adults at risk of cognitive decline (Figure 1). All participants from the CT and AC groups were required to attend a total of 39 one-hour training sessions over thirteen weeks in groups of four to eight. For both the CT and AC groups, each participant was assigned a laptop, a headset, and a mouse that were used for performing the cognitive exercises. They used the same laptop for their entire training. All the training sessions were conducted in a quiet and well-lit room in our laboratory. A research assistant was present in each training session to keep track of their attendance and address any questions pertaining to the task instruction raised by the participants. These research assistants were also responsible for conducting the posttraining assessments.

### 2.5. Outcome Measures

To capture training-associated changes in cognitive function, several cognitive measures on sustained attention, working memory, and memory were administered to all the older adults before and after completing the cognitive training.

#### 2.5.1. Sustained Attention

The Digit Vigilance Test is a measure of vigilance and sustained attention that requires participants to cross out a target number, as fast as they can, throughout the page of digits mixed with other numbers. The numbers are randomly dispersed throughout the page, which adds to the difficulty of identifying the target numbers. Both the reaction time and number of errors are recorded. In our study, only the reaction time was included for statistical analysis. The Seashore Rhythm Test taps an individual's ability to discriminate between “similar” and “dissimilar” pairs of rhythms and demands auditory attention. Participants listen to one pair of musical beats at a time and are asked to judge whether each pair of musical beats is “similar” or “different” right after its presentation. A total of 30 pairs of rhythmic beats will be administered. The total number of accurately identified items is recorded.

#### 2.5.2. Working Memory

The Digit Span Test of the Wechsler Memory Scale, Third Edition (WMS-III), is a measure of auditory working memory. Subjects are instructed to repeat the strings of digits in the same (forward sequence) and reverse (backward sequence) order that is verbally presented by the examiner. The level of difficulty increases as the length of the string of the digits increases. The WMS-III Visual-Spatial Span Test assesses the visual-spatial working memory and requires the subjects to touch or point at the blocks in the same (forward sequence) and reverse (backward sequence) sequences as demonstrated by the examiner.

#### 2.5.3. Memory

The Logical Memory Subtests of the WMS-III were used to measure verbal memory. Subjects are required to recall two stories that are verbally presented by the examiner immediately and after a delay. Following the delayed recall trials, a forced-choice “Yes/No” recognition test is also administered. The Family Pictures Subtest of the WMS-III measures visual-spatial memory. The participants were shown four pictures, one at a time, and were asked to recall the details of each picture.

### 2.6. Statistical Analysis

Prior to the statistical analyses, an independent *t*-test was conducted to examine if any between-group differences (CT and AC) in the demographic variables (age, gender composition, and levels of education), general intellectual abilities (scores on TONI-III), cognitive processing speed (processing speed index on the Wechsler Adult Intelligence Scale, Third Edition (Chinese version)), cognitive status (scores on the Cantonese version of MoCA), and depression (scores on the Geriatric Depression Scale) were present.

A 2 (Training Groups: CT and AC) × 2 (Time-Points: Pretraining and Posttraining) Repeated Measures ANOVA was used to examine the training effect on sustained attention, working memory, and memory, in Chinese older adults at risk of cognitive decline. Post hoc comparisons (paired sample *t*-tests) were carried out for each significant interaction effect to further clarify the effect of cognitive training on a specific outcome measure.

## 3. Results

### 3.1. Participants' Characteristics

Our final sample consisted of 209 older adults aged 60 to 88 years (164 females and 45 males; age: M = 70.1 years; SD = 6.38 years) who successfully completed the pre- and posttraining assessment, of which 109 older adults were randomly assigned to the CT group (87 females and 22 males; age: M = 70.1 years; SD = 6.21 years) and 100 older adults were in the AC group (77 females and 23 males; age: M = 70.0 years; SD = 6.60 years) (Table 1).

Participants from the CT and AC groups were matched for their demographic characteristics (age, gender composition, and years of education), cognitive processing speed, and depression scores (all *P* > .05).

### 3.2. Training-Associated Changes in Cognitive Functioning

#### 3.2.1. Sustained Attention

Seashore Rhythm Test and Digit Vigilance Test are the indicators of the auditory and visual attention. A 2 (Training Groups: CT and AC) × 2 (Time-Points: Pre- and Posttraining) Repeated Measures ANOVA only revealed significant interaction effect on the Seashore Rhythm Test, *F*(1,207) = 5.054, *P* = .026, but not on the reaction time of the Digit Vigilance Test, *F*(1,207) = .046, *P* = .831 (Table 2). Post hoc comparison indicated that only the CT group demonstrated significant improvement in the Seashore Rhythm Test following the training, *t*(108) = −3.707, *P* < .000, whereas the AC group did not show any significant change in their scores but simply maintained a similar level of performance on the Seashore Rhythm Test upon completion of the training, *t*(99) = −.796, *P* = .428.

#### 3.2.2. Working Memory

Digit and Visual-Spatial Span assess the verbal and visual-spatial attention and working memory, respectively. The two-way Repeated Measures ANOVA only identified significant interaction effects on the Total Digit Span, *F*(1,207) = 6.473, *P* = .012, as well as the Total Visual-Spatial Span, *F*(1,207) = 5.047, *P* = .026, which represent auditory and visual-spatial attention and working memory (Table 2).

Post hoc comparisons showed that the CT group exhibited significant increases in the Total Digit Span, *t*(108) = −4.119, *P* = .000, and Total Visual-Spatial Span, *t*(108) = −3.835, *P* = .000, after three months of cognitive training. In contrast, the AC group did not show any statistically significant increase or decrease in the Total Digit Span, *t*(99) = −.121, *P* = .904, and Total Visual-Spatial Span, *t*(99) = −.552, *P* = .582.

#### 3.2.3. Memory (Immediate and Delayed Recall)

Both immediate and delayed recall trials of WMS-III subtests of Logical Memory and Family Picture were chosen to examine the training effect on auditory and verbal memory, respectively. Neither the main effects nor interaction effects on the immediate and delayed recall trials of WMS-III Logical Memory and Family Picture were significant (all *P* > .05) (Table 2).

### 3.3. Correlation between Cognitive Status and Training Effect

To explore whether levels of cognitive status would have been associated with different training outcomes, Pearson correlational analyses were carried out between the baseline general cognitive statuses (as measured by MoCA) and the pre- versus posttraining changes in cognitive measures among the older participants from the CT group. None of the pre- versus posttraining changes in cognitive variables (including attention, working memory, and cognitive processing speed) was significantly correlated with the initial general cognitive statuses of the participants from the CT group (all *P* > .05).

## 4. Discussion

This study used a longitudinal design to examine the specific effect of planned experience on cognitive functioning. Consistent with our* a priori* hypothesis, behavioral changes are specific to the types of experience induced to the brain. In this study, only attention and working memory, but not verbal and visual-spatial memory, showed an improvement after the 13-week systematic cognitive training of these cognitive domains. This pattern of finding is consistent with the prediction set forth by experience-dependent neural plasticity model. The specific improvement in attention and working memory cannot be attributed to other demographic, cognitive, or emotional variables because the CT and AC groups were matched on these dimensions. These training-induced modality-specific improvements in attention and working memory performance have underscored the importance of designing a tailor-made experience as to induce experience-specific changes in cognitive functions (e.g., [2–7, 49, 50]). Perhaps future research can consider exploring the potential of cognitive intervention that is targeted at training a specific cognitive domain or even a specific modality. For example, it is well-documented that atrophy of the medial temporal lobe (especially in the hippocampus) may explain the aging-related memory impairment [24–26]. Based on the findings of this study, planned cognitive training inducing learning and memory consolidation may lead to experience-specific neural plastic changes in the hippocampus leading to long-term memory enhancement. Furthermore, while memory is the major cognitive domain being affected in people suffering from Alzheimer's disease, training targeted at verbal and visual-spatial memory may be beneficial in slowing the memory decline.

The nonsignificant training effect on sustained attention, measured by the Digit Vigilance Test, is inconsistent with previous findings [36]. We speculated that the discrepant findings could relate to the use of different age cohorts between ours and previous studies. Literature has pointed out that normal aging is associated with widespread neuronal and synaptic atrophy [12] and physiological degradation [51], which contribute to age-related blunting of neuroplastic responses in an aging brain [49].

On the CT group, our correlational analyses showed that their initial general cognitive status was not significantly associated with any of the pre- versus posttraining changes in cognitive variables of interests (including attention, working memory, and memory), indicating that older adults who were considered to be at greater risk of cognitive decline could capitalize on the cognitive training to a similar extent and their initial cognitive status may not confine the neural plastic potential of their aging brains. Considering that our study specifically targeted older adults who scored within the restricted range of MoCA (i.e., 19 to 26), it remains unclear whether the significant correlation identified in our study can be generalized to the elderly population who scored in the normal range, so it is worthwhile to look into this unaddressed question in the future.

The findings of this study could be complemented by neuroimaging data that can inform the neural changes associated with the observed behavioral improvement. Future research could investigate whether cognitive training can trigger changes at both the behavioral and neural levels, which could definitely advance our understanding of the mechanisms underlying the training effects. Future studies can probe into the temporal window during which the transition from the neural plastic changes to behavioral changes can be captured. Previous reports have suggested the importance of understanding the generalization of the training effect to activities of daily living (ADL) [52] and other untrained cognitive domains [53]. Unfortunately, we were unable to test for the generalization of the training effects beyond the laboratory setting. It is worthwhile to devote the research effort to elucidate the mechanism underlying the training-transfer effects in order to augment the beneficial effect of cognitive training brought to older adults. While the current study did not intend to inspect the potential impact of educational background on the training effect and hence matched for older participants' level of education, our previous study did report that the less educated older adults were more likely to gain from the cognitive training than their better educated peers [54]. Future investigation can gauge the potential role of educational background and possibly other factors as to maximize the extent to which the older adults can take advantage of the cognitive training.

## 5. Conclusion

The aging of populations is a pressing issue worldwide. Cortical degeneration accompanied by aging, coupled with cognitive deterioration, has placed a heavy socioeconomic burden on the health care system. Increasing research has evidenced that our brain retains a capacity to change in response to experience until late adulthood. Our findings shed insight into the potential of implementing cognitive training for older adults at risk of cognitive decline and provided substantial support that the neural plastic potential continues until older age. Most importantly, this study has provided strong evidence for the potential application of the experience-induced neuroplasticity model to develop cost-effective strategies that can potentially slow down the rate of cognitive decline associated with aging.

## Figures and Tables

**Table 1:** Comparison between the Cognitive Training and Active Control Groups on their demographic characteristics, cognitive processing speed, and depression scores at baseline.

Variables	Cognitive Training (*n* = 109)	Active Control (*n* = 100)	Test of group differences
	M (SD)	M (SD)	*P* value
Age (years)	70.1 (6.21)	70.0 (6.60)	.953
Years of education	8.71 (3.84)	9.49 (4.44)	.173
Gender composition	87 F : 22 M	77 F : 23 M	.736
TONI-III	16.6 (5.34)	17.7 (6.64)	.218
MoCA	23.6 (1.88)	23.8 (1.97)	.494
WAIS-III PSI	71.0 (22.40)	72.3 (22.74)	.677
GDS	4.61 (2.67)	4.31 (2.41)	.403

Note: only the demographic characteristics of the Cognitive Training and Active Control Groups were listed here. TONI-III = Test of Nonverbal Intelligence (Third Edition) (total raw score); MoCA = Montreal Cognitive Assessment, Hong Kong version (Total Score); WAIS-III PSI = Wechsler Adult Intelligence Scale, Third Edition Processing Speed Index (cumulative raw scores of the Digit-Symbol Coding subtest and Symbol Search subtest); GDS = Geriatric Depression Scale; level of significance: *P* < .05.

**Table 2:** Results from the 2 (Training Groups: Cognitive Training and Active Control) × 2 (Time: Pre- and Posttraining) Repeated Measures ANOVA.

Cognitive domain	Raw scores									
	Cognitive Training (*n* = 109)				Active Control (*n* = 100)				Repeated-Measures ANOVA for Training Group-by-Time Interaction	
	Baseline		After 13 weeks of training		Baseline		After 13 weeks of training			
	Mean	SD	Mean	SD	Mean	SD	Mean	SD	*F* (df = 1, 207)	*P* value
Sustained attention										
Digit Vigilance Test	492.53	152.51	460.53	123.93	482.38	114.23	447.13	116.58	.046	.831
Seashore Rhythm Test	**19.80**	**3.84**	**21.13**	**3.70**	**20.73**	**3.60**	**20.98**	**3.57**	**5.054**	**.026** ^*^
Working memory										
Digit Span										
Total	**19.16**	**3.77**	**20.00**	**3.64**	**19.99**	**3.95**	**20.02**	**4.41**	**6.473**	**.012** ^*^
Visual Spatial Span										
Total	**13.11**	**3.06**	**14.04**	**2.91**	**13.44**	**2.97**	**13.58**	**2.91**	**5.047**	**.026** ^*^
Memory										
WMS-III Logical Memory										
Immediate recall	26.21	10.43	30.31	10.68	27.14	9.64	32.26	10.88	.933	.335
Delayed recall	14.81	7.46	18.24	7.73	15.56	7.25	19.75	8.37	.913	.340
WMS-III Family Pictures										
Immediate recall	27.39	10.96	29.20	10.57	27.18	11.24	29.19	11.60	.027	.869
Delayed recall	27.17	10.66	28.57	10.17	26.04	11.77	27.81	11.94	.089	.765

Level of significance: ^*^ *P* < .05.
